# Dynamic changes in coagulation parameters and correlation with disease severity and mortality in patients with COVID-19

**DOI:** 10.18632/aging.203052

**Published:** 2021-05-24

**Authors:** Wei Xu, Ling Fei, Chen Lu Huang, Wei Xia Li, Xu Dong Xie, Qiang Li, Liang Chen

**Affiliations:** 1 Department of Liver Diseases, Shanghai Public Health Clinical Center, Fudan University, Shanghai 201508, China; 2 Department of Infectious Diseases, Shanghai Public Health Clinical Center, Fudan University, Shanghai 201508, China

**Keywords:** COVID-19, SARS-CoV-2, coagulation, disease severity, mortality

## Abstract

Objective: This study aimed to describe the dynamic changes of coagulation parameters and evaluate the relationship between longitudinal coagulation parameters abnormalities and prognosis of COVID-19 patients.

Methods: We performed a retrospective study of 1131 COVID-19 patients. Longitudinal coagulation parameters and clinical outcomes were analyzed.

Results: Abnormal coagulation parameters were observed in patients with COVID-19, both at hospital admission (INR 2.3%, PT 7.9%, APTT 15.4%, TT 0.9%, FDP 2.3%, D-dimer 19.7%) and peak hospitalization (INR 4.8%, PT 13.4%, APTT 25.6%, TT 2.7%, FDP 10.4%, D-dimer 31.5%). Compared with non-severe patients with COVID-19, severe patients had a slightly higher INR, PT, APTT, whereas remarkably higher FDP and D-dimer (*p* < 0.05). On multivariate analysis, age > 60 years, male, obesity, comorbidity, abnormal D-dimer on hospital admission, and abnormal peak hospitalization PT, APTT, FDP and D-dimer were associated with COVID-19 severity. The extreme coagulation parameters abnormalities (PT > 16s, FDP > 50 ug/ml, and D-dimer > 5 ug/ml) were associated with a significantly higher mortality.

Conclusion: Longitudinal coagulation parameters abnormalities are common in patients with COVID-19, and associated with disease severity and mortality. Monitoring coagulation parameters is advisable to improve the management of patients with COVID-19.

## INTRODUCTION

Coronavirus disease 2019 (COVID-19), which is caused by the severe acute respiratory syndrome coronavirus 2 (SARS-CoV-2) [1], is a new type of infectious respiratory disease, and has become an ongoing global pandemic with a profound impact on society and the global economy. Since November 2019, the outbreak of COVID-19 has influenced more than 200 countries, areas or territories worldwide. As of 22 November 2020, there have been over 57.8 million cases and 1.3 million deaths reported globally since the start of the pandemic [2].

Although COVID-19 is well known for causing substantial respiratory pathology, it can also result in several extrapulmonary manifestations [3]. Nearly 20% of COVID-19 patients present coagulation abnormalities, which occur more commonly in the severe and critically ill cases [4]. Coagulopathy has been reported in up to 50% of severe COVID-19 patients, in whom disseminated intravascular coagulation (DIC) has been reported in more than 70% of the patients [5]. Moreover, coagulopathy appears to be associated with the clinical outcomes of COVID-19 patients. A meta-analysis showed that early coagulation tests can predict higher risk stratification and poorer prognosis in patients with COVID-19 [6]. Tang et al. reported that D-dimer and prothrombin time were positively correlated with 28-day mortality of patients with COVID-19 [7]. Yao et al. also reported that D-dimer levels correlate with disease severity and are a reliable prognostic marker for in-hospital mortality in patients admitted for COVID-19 [8].

However, most previous studies described the coagulopathy at hospital admission, the dynamic changes of coagulation parameters during the hospitalization are rare. In addition, few studies have focused on the correlation between abnormal peak hospitalization coagulation parameters and the clinical outcomes of patients with COVID-19. In this study, we aimed to describe the full range of coagulation parameters alterations during the hospital stays and evaluate the relationship between longitudinal coagulation parameters abnormalities and the clinical outcomes of hospitalized patients with COVID-19.

## METHODS

### Participants

A total of 1,131 patients with COVID-19 admitted to Shanghai Public Health Clinical Center, Shanghai, China, between January 20th, 2020 and November 10th,2020, were retrospectively analyzed. Shanghai Public Health Clinical Center is a tertiary teaching hospital, and the only designated hospital for adult patients with COVID-19 in Shanghai, China. Among 1,131 enrolled patients, 13 patients were categorized into severe group, and 1,118 patients were categorized into non-severe group at hospital admission. Among 1,118 non-severe patients at hospital admission, 23 patients progressed to severe COVID-19 during the hospital stays, and therefore were switched to severe group. By the end of follow-up (November 10th, 2020), a total of 36 severe patients and 1095 non-severe patients were identified in this study. Among 36 severe patients with COVID-19, 7 died, and 29 were discharged with recovery.

### Diagnostic criteria

Patients with COVID-19 were confirmed based on the detection of SARS-CoV-2 RNA in nasopharyngeal or throat swab specimens using the polymerase chain reaction (PCR) method [9]. Severe patients were defined as any one of the following [9]: (1) Respiratory rates ≥ 30/min; (2) Resting oxygen saturation ≤ 93%; (3) Oxygenation index ≤ 300 mmHg; (4) Require mechanical ventilation; (5) shock; (6) Combined with other organ failures and needed the intensive care unit (ICU) admission. Bleeding events were graded according to the modified World Health Organization (WHO) grading system, and included gastrointestinal bleeding, hemoptysis, oral mucosa bleeding, epistaxis, internal bleeding, pulmonary hemorrhage, bleeding from multiple cannulation sites, and intracranial hemorrhage [10].

### Data collection

All data were extracted from the electronic records of Shanghai Public Health Clinical Center. Sociodemographic data were obtained, including age, sex, body mass index (BMI), and underlying diseases. The laboratory tests, chest CT scans, medical management information, clinical outcomes, and length of hospitalization were collected. The following coagulation parameters were recorded at hospital admission and during the hospital stays: prothrombin time (PT), international normalized ratio (INR), activated partial thromboplastin time (APTT), thrombin time (TT), fibrinogen degradation products (FDP), and D-dimer. The coagulation parameters measurements were performed on Stago~(STA)RMAX coagulation analyzers and using the original reagents (Startec Diagnostics Co., LTD, France). Coagulation parameters abnormalities were defined as per Shanghai Public Health Clinical Center laboratory reference range standards: INR > 1.2, PT > 14s, APTT > 43s, TT > 21s, FDP > 5 ug/ml, and D-dimer > 0.5 ug/ml. In this study, the levels of IL-6 in most of patients were below the low limitation of the human cytokine kit II (Raisecare Ltd, Qingdao, China), and were showed as “0” values in the reports of Shanghai Public Health Clinical Center laboratory. Therefore, in this study, the “0” values mean the levels of IL-6 were below the low limitation of detection, rather than the actual test values.

### Statistical analysis

Normal distribution variables were presented using mean and standard deviations, non-normal distribution continuous variables were presented using median and interquartile ranges (IQRs), and qualitative data were presented using frequency distribution. For the comparison of quantitative data between two groups, we used the Student’s *t*-test for normal distribution variables, and the non-parametric Mann-Whitney-test for non-normal distribution variables. For the comparison of qualitative variables, we used the chi-squared test. Clinical outcomes were modeled using the coagulation parameters at hospital admission and their peak during hospitalization. The multivariate logistic regression analysis was performed to adjust for age, gender, obesity, comorbidity, and the coagulation factors. We performed the survival estimates using the Kaplan–Meier method, comparing the survival curves according to the coagulation parameters between the groups.

### Ethics approval and consent to participate

The study was approved by the Clinical Research Ethics Committee of the Shanghai Public Health Clinical Center. The study was conducted in accordance with the principles of the Helsinki declaration of 1975, as revised in 1983.

## RESULTS

### Demographic and clinical characteristics of the study population

The demographic and clinical characteristics of the study population are shown in Table 1. The median age was 36 years (IQR, 26–50), 690 patients (61%) were men, 320 patients (28.3%) had obesity, and 202 patients (17.9%) had comorbidity. The median levels of white blood count (WBC), lymphocyte, platelet, C-reactive protein (CRP), erythrocyte sedimentation rate (ESR), interleukin-6 (IL-6), and interleukin-8 (IL-8) were 5.7 × 10^9^/L (IQR, 4.5–7.1), 1.5 × 10^9^/L (IQR, 1.1–2.0), 220 × 10^9^/L (IQR, 175–265), 0.5 mg/L (IQR, 0.5-3.8), 26 mm/h (IQR, 10–47), 0 pg/ml (IQR, 0–0), and 1.0 pg/ml (IQR, 0.3–2.5), respectively. Compared with non-severe patients, severe patients had higher age (median, 64 *vs* 35 years, *p* < 0.001), more common male (77.8% *vs* 60.5%, *p* = 0.010), obesity (50% *vs* 27.6%, *p* = 0.001), and comorbidity (69.4% *vs* 16.2%, *p* < 0.001). Patients in severe group had a lower lymphocyte (0.8 *vs* 1.6 × 10^9^/L, *p* < 0.001) and platelet (165 *vs* 222 × 10^9^/L, *p* < 0.001), but notably higher CRP (36.4 *vs* 0.5 mg/L, *p* < 0.001), ESR (49 *vs* 25 mm/h, *p* < 0.001), IL–6 (40.5 *vs* 0 pg/ml, *p* < 0.001), and IL-8 levels (8.4 *vs* 0.8 pg/ml, *p* < 0.001) than patients in non-severe group (Table 1).

**Table 1:** Demographic and clinical characteristics of the study population.

	**Total (*n* = 1131)**	**Non-severe (*n* = 1095)**	**Severe (*n* = 36)**	***p*-value**
Age (years)	36 (26–50)	35 (26–49)	64 (50–73)	< 0.001
Male, *n* (%)	690 (61%)	662 (60.5%)	28 (77.8%)	0.010
Obesity, *n* (%)	320 (28.3%)	302 (27.6%)	18 (50%)	0.001
Comorbidity, *n* (%)	202 (17.9%)	177 (16.2%)	25 (69.4%)	< 0.001
Hypertension	126 (11.1%)	107 (9.8%)	19 (52.8%)	< 0.001
Diabetes	58 (5.1%)	50 (4.6%)	8 (22.2%)	< 0.001
Laboratory findings				
WBC (10^9^/L)	5.7 (4.5–7.1)	5.7 (4.5–7.1)	5.9 (3.8–7.3)	0.503
LYMP (10^9^/L)	1.5 (1.1–2.0)	1.6 (1.2–2.0)	0.8 (0.5–1.1)	< 0.001
Platelet (10^9^/L)	220 (175–265)	222 (177–266)	165 (123–206)	< 0.001
CRP (mg/L)	0.5 (0.5–3.8)	0.5 (0.5–2.9)	36.4 (9.5–81.9)	< 0.001
ESR (mm/h)	26 (10–47)	25 (10–46)	49 (37–89)	< 0.001
IL-6 (pg/ml)	0 (0–0)	0 (0–0)	40.5 (24.6–80.4)	< 0.001
IL-8 (pg/ml)	1.0 (0.3–2.5)	0.8 (0.2–2.3)	8.4 (3.2–25.6)	< 0.001
Prophylactic Anticoagulation and Thrombotic Complications				
Anticoagulation	55 (4.9%)	20 (1.8%)	35 (97.2%)	< 0.001
Plasma transfusion	17 (1.5%)	2 (0.2%)	15 (41.7%)	< 0.001
VTE	9 (0.8%)	2 (0.2%)	7 (19.4%)	< 0.001
PE	4 (0.35%)	0	4 (11.1%)	< 0.001
Bleeding events	2 (0.2%)	0	2 (5.6%)	< 0.001

Abbreviations: WBC: white blood count; LYMP: lymphocyte; CRP: C-reactive protein; ESR: erythrocyte sedimentation rate; VTE: venous thromboembolism; PE: Pulmonary embolism. The *p* values indicate differences between severe group and non-severe group.

### Prophylactic anticoagulation, thrombotic complications, and bleeding events

During the hospital stays, 55 patients (4.9%) received prophylactic anticoagulation, and 17 patients (1.5%) received plasma transfusion. Prophylactic anticoagulation therapies were more frequently used in severe patients compared with non-severe patients (97.2% *vs* 1.8%, *p* < 0.001). Patients on prophylactic anticoagulation had higher age, more common comorbidity, higher D-dimer and FDP levels compared with those without anticoagulation (all *p* < 0.05). Despite prophylactic anticoagulation, we found a radiographically confirmed venous thromboembolism (VTE) rate of 13.8%, pulmonary embolisms (PE) rate of 11.1%, in severe patients with COVID-19 (Table 1). During the hospital stays, 3 patients (8.3%) in severe group and 8 patients (0.7%) in non-severe group developed bleeding events (Table 1).

### Usage and dosage of prophylactic anticoagulation

The usage and dosage of prophylactic anticoagulation are shown as following: (1) low molecular weight heparin (LMWH), 5,000 U daily, subcutaneous injection, 39 patients; (2) enoxaparin, 4,000 IU daily, subcutaneous injection, 10 patients; (3) warfarin, 2.5mg daily, oral administration, 6 patients. Once patients were confirmed with PE/ VTE, prophylactic anticoagulation therapies were replaced with therapeutic anticoagulation (LMWH, 5,000 U twice daily, or enoxaparin 4,000 IU twice daily).

### Coagulation parameters between severe and non-severe patients on hospital admission

**Figure 1:**
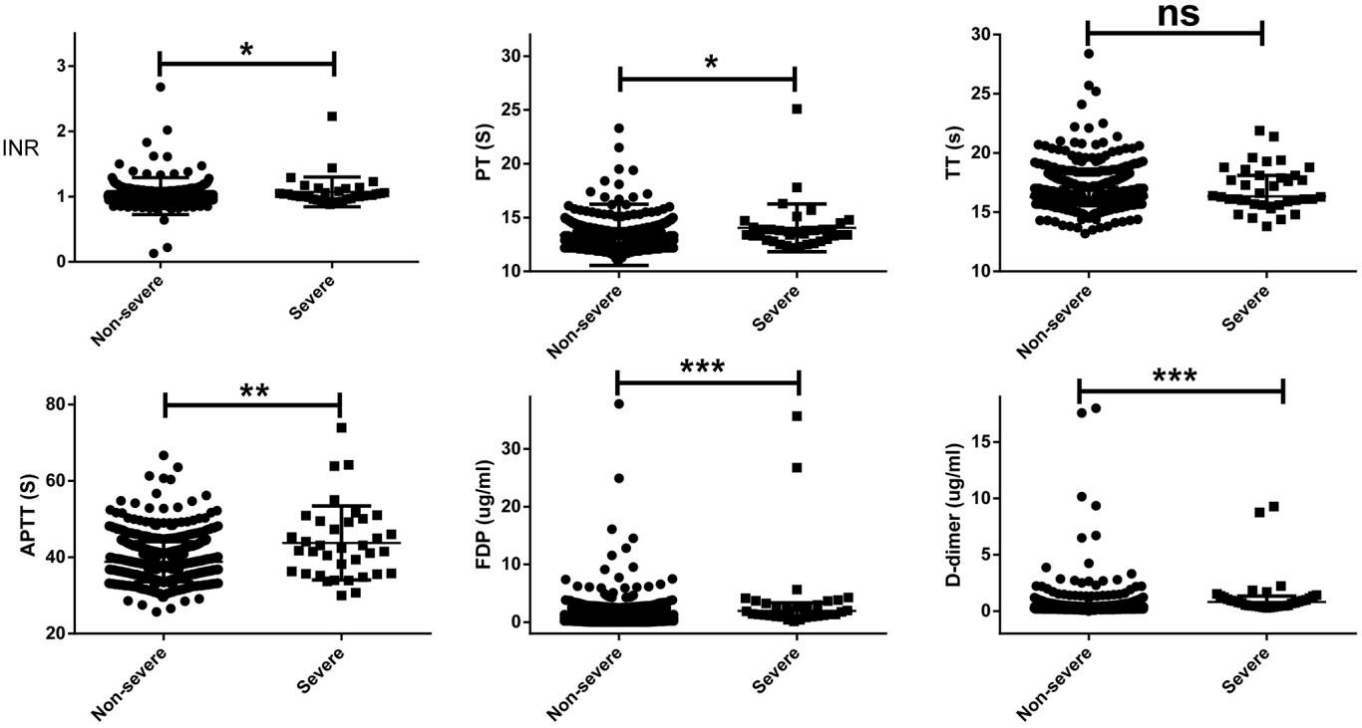
**A scatter plots of the coagulation values on hospital admission between severe and non-severe patients.** The severe patients had a slightly higher INR (1.02 *vs* 0.99, *p* = 0.016), PT (13.6s *vs* 13.2s, *p* = 0.019), APTT (42.1s *vs* 38.4s, *p* = 0.019), whereas remarkably higher FDP (2.03 *vs* 0.65 ug/ml, *p* < 0.001) and D-dimer (0.87 *vs* 0.27 ug/ml, *p* < 0.001) than non-severe patients.

The differences in coagulation parameters between severe and non-severe patients on hospital admission are shown in Table 2. On hospital admission, the severe patients had a slightly higher INR (1.02 *vs* 0.99, *p* = 0.016), PT (13.6s *vs* 13.2s, *p* = 0.019), APTT (42.1s *vs* 38.4s, *p* = 0.019), whereas remarkably higher FDP (2.03 *vs* 0.65 ug/ml, *p* < 0.001) and D-dimer (0.87 *vs* 0.27 ug/ml, *p* < 0.001) than non-severe patients. Abnormal INR (11.1% *vs* 2.0%, *p* < 0.001), PT (30.6% *vs* 7.1%, *p* < 0.001), APTT (47.2% *vs* 14.3%, *p* < 0.001), TT (5.6% *vs* 0.7%, *p* = 0.002), FDP (13.9% *vs* 1.9%, *p* < 0.001), and D-dimer (69.4% *vs* 18.1%, *p* < 0.001) were commonly observed in severe patients, compared with non-severe patients. A scatter plots of the coagulation values between severe and non-severe patients on hospital admission are shown in Figure 1.

**Table 2:** Coagulation parameters between severe and non-severe patients on hospital admission.

	**Total**	**Non-severe**	**Severe**	***p*-value**
Number	1131	1095	36	
INR	0.99 (0.95–1.04)	0.99 (0.95–1.04)	1.02 (0.96–1.08)	0.016
Abnormal (> 1.2)	26 (2.3%)	22 (2.0%)	4 (11.1%)	< 0.001
PT (s)	13.0 (13.0–14.0)	13.2 (12.8–13.7)	13.6 (12.9–14.1)	0.019
Abnormal (> 14s)	89 (7.9%)	78 (7.1%)	11 (30.6%)	< 0.001
APTT (s)	38.5 (35.7–41.5)	38.4 (35.7–41.5)	42.1 (35.7–49.4)	0.001
Abnormal (> 43s)	174 (15.4%)	157 (14.3%)	17 (47.2%)	< 0.001
TT (s)	16.3 (15.7–17.1)	16.3 (15.7–17.1)	16.4 (15.8–18.1)	0.194
Abnormal (> 21s)	10 (0.9%)	8 (0.7%)	2 (5.6%)	0.002
FDP (ug/ml)	0.70 (0.20–1.40)	0.65 (0.22–1.37)	2.03 (1.13–3.68)	< 0.001
Abnormal (> 5 ug/ml)	26 (2.3%)	21 (1.9%)	5 (13.9%)	< 0.001
D-dimer (ug/ml)	0.28 (0.20–0.44)	0.27 (0.20–0.42)	0.87 (0.47–1.41)	< 0.001
Abnormal (> 0.5 ug/ml)	223 (19.7%)	198 (18.1%)	25 (69.4%)	< 0.001

Abbreviations: INR: international normalized ratio; PT: prothrombin time; APTT: activated partial thromboplastin time; TT: thrombin time; FDP: fibrinogen degradation products. The *p* values indicate differences between severe group and non-severe group.

### Hospital admission *vs* peak hospitalization coagulation parameters

The prevalence and severity of abnormal coagulation parameters during the hospitalization are shown in Table 3. Abnormal coagulation parameters were observed in patients with COVID-19, both at hospital admission (INR 2.3%, PT 7.9%, APTT 15.4%, TT 0.9%, FDP 2.3%, DD 19.7%) and peak hospitalization (INR 4.8%, PT 13.4%, APTT 25.6%, TT 2.7%, FDP 10.4%, DD 31.5%). Most patients had mild coagulation laboratory parameters changes at hospital admission (INR 1–2 ULN, 92.4%; PT prolonged 1–3s, 89.9%; APTT prolonged 1-3s, 63.8%; TT prolonged 1–3s, 70%; FDP 1–5 ULN, 73.1%; D-dimer 1–5 ULN, 91.9%), as well as peak hospitalization (INR 1–2 ULN, 79.6%; PT prolonged 1–3s, 87.4%; APTT prolonged 1-3s, 57.6%; TT prolonged 1–3s, 70%; FDP 1-5 ULN, 74.6%; D-dimer 1–5 ULN, 80.1%). The severe coagulation laboratory changes were relatively infrequently observed at hospital admission (INR > 3 ULN, 3.8%; PT prolonged > 6s, 4.5%; APTT prolonged > 6s, 22.4%; TT prolonged > 6s, 10%; FDP > 10 ULN, 15.4%; D-dimer > 10 ULN, 4.5%), as well as peak hospitalization (INR > 3 ULN, 14.8%; PT prolonged > 6s, 6.0%; APTT prolonged > 6s, 26.2%; TT prolonged > 6s, 16.7%; FDP > 10 ULN, 16.1%; D-dimer > 10 ULN, 12.4%).

**Table 3:** Hospital admission vs peak hospitalization coagulation parameters in 1131 patients.

		**Hospital admission**	**Peak Hospitalization**
INR	Abnormal (> 1.2)	26 (2.3%)	54 (4.8%)
	1–2 ULN	24 (92.4%)	43 (79.6%)
	2–3 ULN	1 (3.8%)	3 (5.6%)
	> 3 ULN	1 (3.8%)	8 (14.8%)
PT (s)	Abnormal (> 14s)	89 (7.9%)	151 (13.4%)
	Prolonged 1–3s	80 (89.9%)	132 (87.4%)
	Prolonged 4–6s	5 (5.6%)	10 (6.6%)
	Prolonged > 6s	4 (4.5%)	9 (6.0%)
APTT (s)	Abnormal (> 43s)	174 (15.4%)	290 (25.6%)
	Prolonged 1–3s	111 (63.8%)	167 (57.6%)
	Prolonged 4–6s	24 (13.8%)	47 (16.2%)
	Prolonged > 6s	39 (22.4%)	76 (26.2%)
TT (s)	Abnormal (> 21s)	10 (0.9%)	30 (2.7%)
	Prolonged 1–3s	7 (70%)	21 (70%)
	Prolonged 4–6s	2 (20%)	4 (13.3%)
	Prolonged > 6s	1 (10%)	5 (16.7%)
FDP (ug/ml)	Abnormal (> 5 ug/ml)	26 (2.3%)	118 (10.4%)
	1–5 ULN	19 (73.1%)	88 (74.6%)
	5–10 ULN	3 (11.5%)	11 (9.3%)
	> 10 ULN	4 (15.4%)	19 (16.1%)
D-dimer (ug/ml)	Abnormal (> 0.5 ug/ml)	223 (19.7%)	356 (31.5%)
	1–5 ULN	205 (91.9%)	285 (80.1%)
	5–10 ULN	8 (3.6%)	27 (7.6%)
	> 10 ULN	10 (4.5%)	44 (12.4%)

Abbreviations: INR: international normalized ratio; PT: prothrombin time; APTT: activated partial thromboplastin time; TT: thrombin time; FDP: fibrinogen degradation products; ULN: upper limit of normal. The ULNs of coagulation parameters were defined as per Shanghai Public Health Clinical Center laboratory reference range standards: INR ≤ 1.2, PT ≤ 14s, APTT ≤ 43s, TT ≤ 21s, FDP ≤ 5 ug/ml, D-dimer ≤ 0.5 ug/ml.

### Coagulation parameters classified by aged/non-aged, obese/non-obese, oncologic/non-oncologic, and diabetic/non-diabetic

We analyzed coagulation parameters abnormalities on hospital admission classified by aged/non-aged, obese/non-obese, oncologic/non-oncologic, and diabetic/non-diabetic in Table 4. The results showed that coagulation parameters abnormalities were commonly observed in patients with age > 60 years, diabetes or malignant tumour compared with patients without the above situations (*p* < 0.05). There is no significant difference between obese patients and non-obese patients in all coagulation parameters abnormalities (*p* > 0.05) (Table 4).

**Table 4:** Coagulation parameters on hospital admission classified by aged/non-aged, obese/non-obese, oncologic/non-oncologic, and diabetic/non-diabetic.

	**Age (years)**			**Obesity**			**Malignant tumour**			**Diabetes**		
	**≤ 60**	**>60**	***p***	**With**	**Without**	***p***	**With**	**Without**	***p***	**With**	**Without**	***p***
	**(*n* = 1003)**	**(*n* = 128)**		**(*n* = 320)**	**(*n* = 811)**		**(*n* = 8)**	**(*n* = 1123)**		**(*n* = 58)**	**(*n* = 1073)**	
Abnormal INR	19 (1.9%)	7 (5.5%)	0.011	5 (1.6%)	21 (2.6%)	0.299	2 (25%)	24 (2.1%)	< 0.001	5 (8.6%)	21 (2.0%)	0.001
Abnormal PT	729 (7.2%)	17 (13.3%)	0.016	15 (4.7%)	74 (9.1%)	0.130	2 (25%)	87 (7.8%)	< 0.001	10 (17.2%)	79 (7.4%)	0.007
Abnormal APTT	145 (14.5%)	29 (22.7%)	0.016	52 (16.3%)	122 (15.0%)	0.612	2 (25%)	172 (15.3%)	< 0.001	24 (41.4%)	150 (14.0%)	< 0.001
Abnormal TT	5 (0.5%)	5 (3.9%)	< 0.001	3 (0.9%)	5 (0.6%)	0.562	1 (12.5%)	9 (0.8%)	< 0.001	3 (5.2%)	7(0.7%)	< 0.001
Abnormal FDP	17 (1.7%)	9 (7.0%)	< 0.001	8 (2.5%)	18 (2.2%)	0.777	2 (25%)	25 (22.3%)	< 0.001	4 (6.9%)	22(2.1%)	0.017
Abnormal D-dimer	161 (16.1%)	62 (48.4%)	<0.001	72 (22.5%)	151 (18.6%)	0.140	4 (50%)	219 (19.5%)	0.031	25 (43.1%)	198 (18.5%)	< 0.001

Abbreviations: INR: international normalized ratio; PT: prothrombin time; APTT: activated partial thromboplastin time; TT: thrombin time; FDP: fibrinogen degradation products.

### Correlations between hematologic parameters and coagulation parameters

The correlation between hematologic parameters and coagulation parameters was analyzed using the Spearman test in Table 5. The results showed that lymphocyte is negatively correlated with APTT (r = –0.13, *p* < 0.001), FDP (r = –0.10, *p* = 0.001), and D-dimer (r = –0.14, *p* < 0.001); CRP is positively correlated with APTT (r = 0.20, *p* < 0.001), FDP (r = 0.30, *p* < 0.001), and D-dimer (r = 0.31, *p* < 0.001); ESR is positively correlated with FDP (r = 0.11, *p* < 0.001) and D-dimer (r = 0.17, *p* < 0.001); IL-6 is positively correlated with PT (r = 0.25, *p* < 0.001), APTT (r = 0.28, *p* < 0.001), FDP (r = 0.42, *p* < 0.001), and D-dimer (r = 0.45, *p* < 0.001); IL-8 is positively correlated with PT (r = 0.23, *p* < 0.001), APTT (r = 0.24, *p* < 0.001), FDP (r = 0.38, *p* < 0.001), and D-dimer (r = 0.40, *p* < 0.001).

**Table 5:** Correlations between hematologic parameters and coagulation parameters on admission.

	**PT**		**APTT**		**FDP**		**D-dimer**		**TT**	
	**r**	***p***	**r**	***p***	**r**	***p***	**r**	***p***	**r**	***p***
WBC	0.02	0.554	–0.04	0.171	0.18	< 0.001	0.11	< 0.001	0.02	0.474
Platelet	0.02	0.447	–0.06	0.097	0.01	0.729	–0.04	0.177	0.05	0.109
LYMP	–0.01	0.830	–0.13	< 0.001	–0.10	0.001	–0.14	< 0.001	0.01	0.763
CRP	0.02	0.492	0.20	< 0.001	0.30	< 0.001	0.31	< 0.001	–0.01	0.690
PCT	0.02	0.578	0.01	0.652	–0.01	0.873	–0.01	0.869	0.01	0.639
ESR	–0.20	0.605	0.04	0.148	0.11	< 0.001	0.17	< 0.001	–0.01	0.938
IL-6	0.25	< 0.001	0.28	< 0.001	0.42	< 0.001	0.45	< 0.001	0.04	0.188
IL-8	0.23	< 0.001	0.24	< 0.001	0.38	< 0.001	0.40	< 0.001	0.03	0.216

Abbreviations: WBC: white blood count; LYMP: Lymphocyte; CRP: C-reactive protein; PCT: procalcitonin; ESR: erythrocyte sedimentation rate; PT: prothrombin time; APTT: activated partial thromboplastin time; FDP: fibrinogen degradation products; TT: thrombin time; r: correlation coefficient.

### Association between coagulation parameters and clinical outcomes

**Figure 2:**
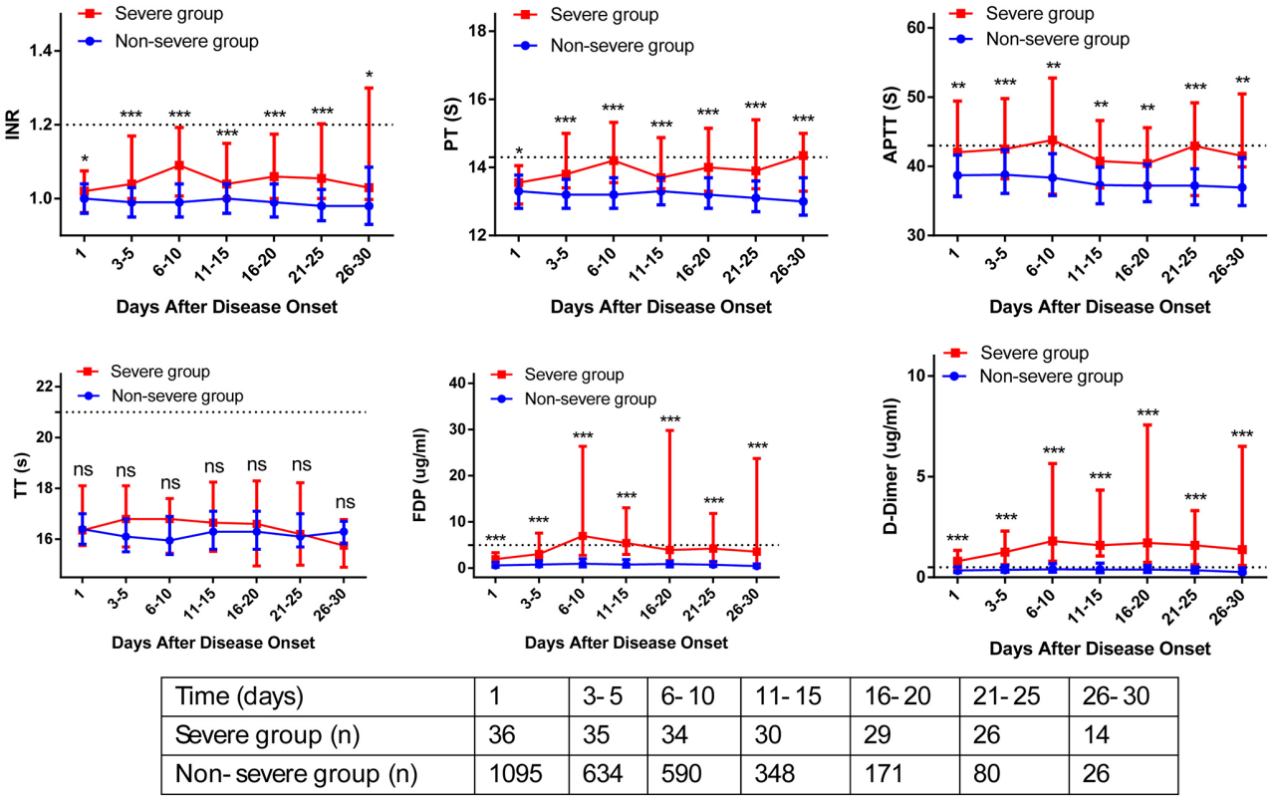
**Dynamic profile of coagulation parameters in patients by severity of COVID-19.** The coagulation parameters in the non-severe group (blue line) and severe group (red line) were analyzed at different time points after hospital admission. The coagulation parameters are shown using median and IQR.

**Figure 3:**
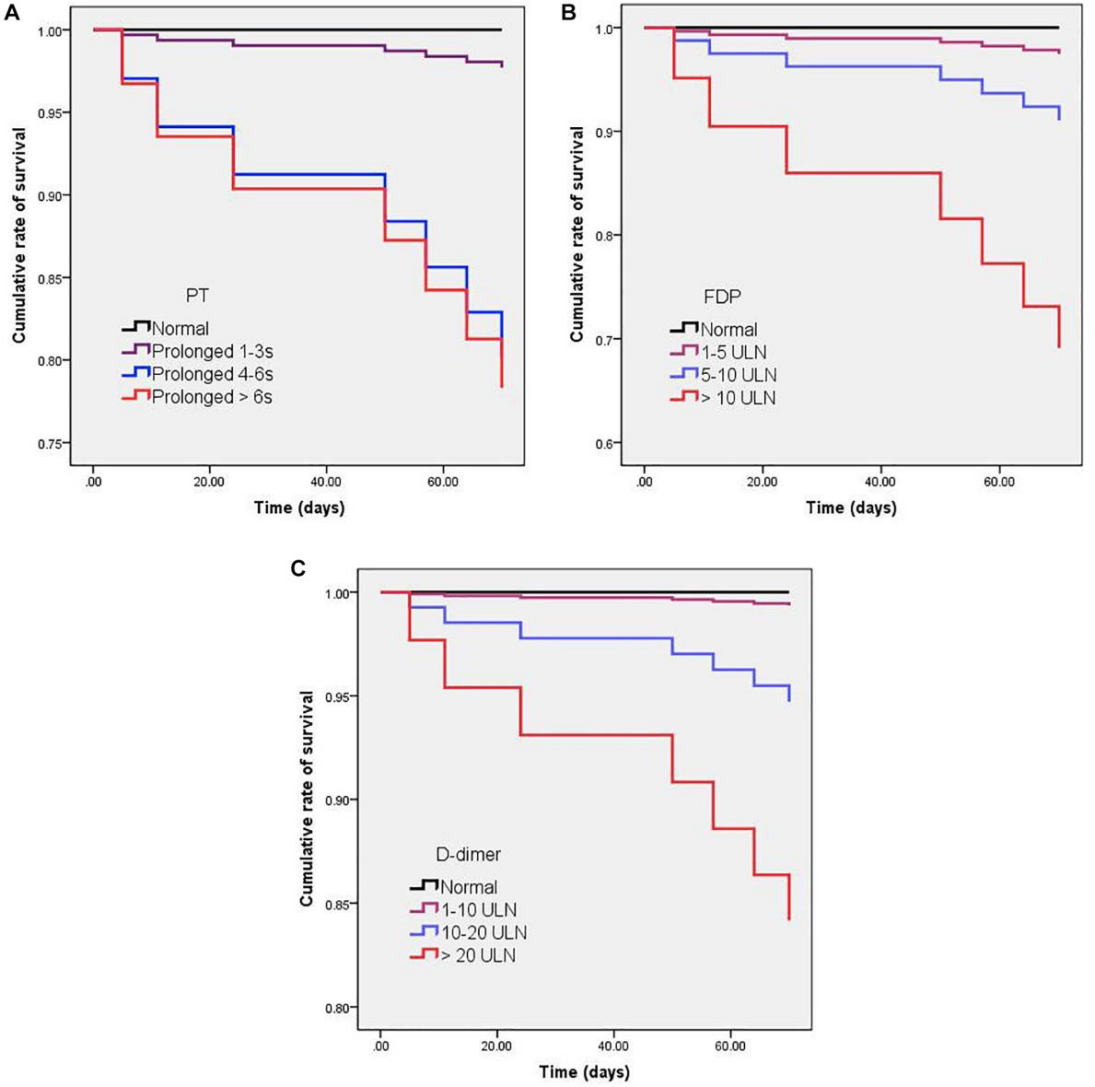
**Kaplan-Meier curves for cumulative rate of survival in patients with different levels of PT (A), FDP (B), and D-dimer (C).** Cox regression analysis showed that PT prolonged > 4s (HR = 90; CI% = 20–404; *p* < 0.001), D-dimer > 10 ULN (HR = 64; CI% = 13–334; *p* < 0.001), and FDP > 10 ULN (HR = 48; CI% = 11–214; *p* < 0.001) were significantly associated with a higher mortality.

The association between admission and peak hospitalization coagulation parameters and clinical outcomes is summarized in Table 6. On multivariate analysis, age > 60 years, male, obesity, comorbidity, abnormal D-dimer on hospital admission, and abnormal peak hospitalization PT, APTT, FDP, and D-dimer were associated with severe COVID-19 (OR > 1; *p* < 0.05). The dynamic profile of coagulation parameters in patients by severity of COVID-19 is illustrated in Figure 2. Compared with non-severe patients, severe COVID-19 patients had markedly higher levels of INR, PT, APTT, FDP, and D-dimer from baseline to 30 days after admission (*p* < 0.05). On multivariate analysis, age > 60 years, obesity, comorbidity, and abnormal peak hospitalization PT (OR = 3.32; 95% CI 1.43–24.94; *p* = 0.001), FDP (OR = 2.63; 95% CI 1.16–5.57; *p* = 0.032), and D-dimer (OR = 3.21; 95% CI 1.32–21.65; *p* = 0.026) were associated with death. Kaplan-Meier curves for cumulative rate of survival during hospitalization in patients with different level of PT (a), FDP (b), and D-dimer (c) are illustrated in Figure 3.

**Table 6:** Association between coagulation parameters and clinical outcomes (Multivariate model).

	**Severe COVID-19**		**Death**	
	**OR (90% CI)**	***p*-value**	**OR (90% CI)**	***p*-value**
Age > 60 years	3.48 (1.33–9.12)	0.011	6.01 (1.78**–**15.27)	0.007
Male	2.85 (1.08–7.53)	0.035	1.60 (0.31–8.26)	0.577
Obesity	3.05 (1.13–8.21)	0.028	1.75 (1.21–4.32)	0.028
Comorbidity	3.93 (1.55–9.95)	0.004	6.51 (2.83–19.32)	< 0.001
Hospital Admission				
Abnormal INR	0.62 (0.09–4.53)	0.638	0.42 (0.02–7.46)	0.515
Abnormal PT	0.53 (0.11–2.65)	0.438	1.17 (0.35–3.46)	0.280
Abnormal APTT	1.36 (0.46–4.02)	0.579	1.67 (0.48–5.18)	0.374
Abnormal TT	0.45 (0.03–7.55)	0.576	2.23 (0.47–8.25)	0.127
Abnormal FDP	2.34 (0.56–9.83)	0.245	0.24 (0.01–2.55)	0.424
Abnormal D-dimer	1.58 (1.16–4.18)	0.035	0.74 (0.07–3.59)	0.612
Peak Hospitalization				
Abnormal INR	0.89 (0.70–5.14)	0.208	0.98 (0.37–18.19)	0.874
Abnormal PT	2.07 (1.18–5.97)	< 0.001	3.32 (1.43–24.94)	0.001
Abnormal APTT	3.51 (1.39–8.85)	0.008	2.33 (0.54–8.46)	0.127
Abnormal TT	2.53 (0.73–8.58)	0.145	0.31 (0.02–5.71)	0.577
Abnormal FDP	2.50 (1.04–6.27)	0.041	2.63 (1.16–5.57)	0.032
Abnormal D-dimer	3.85 (1.42–8.46)	0.012	3.21 (1.32–21.65)	0.026

Abbreviations: OR: odds ratio; CI: confidence interval; INR: international normalized ratio; PT: prothrombin time; APTT: activated partial thromboplastin time; TT: thrombin time; FDP: fibrinogen degradation products.

### Further analyzed patients had significant increase in PT and D-dimer

In this study, 151 patients had increased PT during the hospital stays, among them, 2 patients developed bleeding events. The 2 patients with bleeding events were severe cases and on anticoagulation. The bleeding rates of patients receiving anticoagulants is 2/55 (3.6%). In our cohort, 44 patients had the significant increase in D-dimer (> 10 ULN), among them, 17 patients (38.6%) had the serious illness, and 5 patients (11.4%) died. Cox regression analysis showed the significant increase in D-dimer (> 10 ULN) was associated with serious illness (HR = 22; CI% = 6-117; *p* < 0.001), and a higher mortality (HR = 64; CI% = 13–334; *p* < 0.001).

## DISCUSSION

This study aimed to describe the dynamic changes of coagulation parameters during hospitalization, and evaluate the association between longitudinal coagulation parameters abnormalities and clinical outcomes of COVID-19 patients. The strength of this study included its large sample size, and the longitudinal coagulation parameters description at admission and during hospitalization. Although the differences in coagulation parameters were not always striking (some moving within the ULN), Figure 2 clearly showed that severe COVID-19 patients had higher levels of coagulation parameters than non-severe patients from baseline to 30 days after admission. Figure 3 clearly showed that peak hospitalization PT, FDP, and D-dimer were associated with mortality of COVID-19 patients.

Previous studies also indicated the association between abnormal coagulation parameters and the poor prognosis of COVID-19 patients [11]. In the study published by Quintana-Diaz et al, non-survivors showed a more than 3.5-fold increase in D-dimer compared with the survivors [12]. Yao et al. reported that median dimer D-in non-survivors was significantly higher than that in survivors [6.21 *vs* 1.02 mg/L, *p* < 0.001], and D-dimer of > 2.14 mg/L predicted in-hospital mortality with a sensitivity of 88.2% and specificity of 71.3% [8]. A meta-analysis showed the risk of mortality was four-fold higher in patients with high D-dimer vs. normal D-dimer (risk ratio 4.11, 95% CI 2.48 – 6.84, *p* < 0.001) and the risk of developing severe disease was two-fold higher in patients with high D-dimer *vs*. normal D-dimer (risk ratio 2.04, 95% CI 1.34 – 3.11, *p* < 0.001) [13].

Though the specific mechanisms are still unclear, SARS-CoV-2 obviously involves potentially deleterious processes in hemostasis/coagulation system. The potential causes of coagulopathy and fibrinolytic disruption in patients with COVID-19 are shown as following: (1) Dysfunctional angiotensin converting enzyme 2 (ACE2). Dysfunction of ACE2 leads to abnormal renin-angiotensin (RAS) system activation, which promotes platelet adhesion and aggregation and enhances the risk of thromboembolism following the invasion of SARS-CoV-2 [14]. (2) Innate immune response. In fact, the regulation of coagulation and innate immunity is intertwined because they share some common pathways in response to viral invasion, such as the function of tissue factor in the initiation of procoagulation and the host immune response [15]. (3) Inflammatory factor storm. Inflammation due to SARS-CoV-2 infection aggravates various proinflammatory cytokines, which increase the expression of tissue factor and von Willebrand factor from endothelial cells and monocytes, promoting platelet aggregation and initiating the clotting cascade [14]. Besides, proinflammatory cytokines can also suppress the synthesis of anticoagulants and fibrinolysis by downregulating thrombomodulin and endothelial protein C receptor and upregulating plasminogen activator inhibitor-1(PAI-1) levels, which will finally activate coagulation cascade and inhibit fibrinolytic reaction [16]. (4) Endothelial cell infection and endotheliitis. Rapidly emerging data are providing insight into how endothelial dysfunction may contribute to the coagulopathy in patients with COVID-19 [17].

In this study, among 36 severe patients, 35 (97.2%) received prophylactic anticoagulation. Despite prophylactic anticoagulation, we found a radiographically confirmed VTE rate of 19.4%, PE rate of 11.1%, in severe patients with COVID-19. Helms et al. also reported that despite anticoagulation, a number of patients with ARDS secondary to COVID-19 developed thrombotic complications, mainly PE (16.7%) [18]. Although this study and other study find clotting events in COVID-19 patients with prophylactic anticoagulation, we declare that anticoagulation helps to reduce the risk of clotting events in severe and critically ill COVID-19 patients. Perhaps the coagulation burden would be even higher if not on the anticoagulation. For example, Cui et al. reported that the overall incidence of VTE was 25% in severe COVID-19 patients without anticoagulation [19]. Llitjos et al. reported the overall rate of VTE was 69% in severe COVID-19 patients [20]. According to a consensus statement for prevention and treatment of VTE associated with COVID-19, all severe and critically ill COVID-19 patients have a high risk of VTE, so anticoagulation is strongly recommended in absence of contraindication [4].

In this study, the incidence rate of bleeding events in patients receiving anticoagulants is 2/55 (3.6%), which is consistent with previous studies. Two groups with patients receiving anticoagulants reported bleeding rates of 0% to 3% [21, 22]. Paranjpe et al. compared systemic anticoagulation *vs* no anticoagulation in 2772 patients with COVID-19, and found no difference in bleeding events (1.9% *vs* 3%; *p* > 0.05) [22]. Tafur et al. analyzed 1496 patients received anticoagulation therapy, and found that the 3-month cumulative incidence rates of major and overall bleeding were 2.1% and 5.1%, respectively [23]. In conclusion, a few reports exist regarding bleeding outcomes for hospitalized patients receiving either prophylaxis or therapeutic anticoagulation, which may reduce venous thrombotic events while slightly increasing bleeding events. Decision making requires a careful balance between these 2 outcomes to maximize net clinical benefit for these patients.

Elevated levels of inflammation-related cytokines, including IL-6, IL-8, and IL-10 were found in patients with COVID-19 [24]. In this study, we found that IL-6 and IL-8 levels are positively correlated with abnormal PT, APTT, FDP, and D-dimer. The results showed the potential of the intimate interconnection with inflammatory disorders, hypercoagulation and excessive immunity following SARS-CoV-2 invasion in dysfunctional coagulation. The stimulation of an immune response and proinflammation activate the coagulation cascade, and blood clotting in turn orchestrates the pathway of an excessive inflammatory response [14].

The limitations of this study are evident. First, retrospective observational cohort study design with inclusion restricted to patients who were hospitalized within a single hospital, and limited access to laboratory, and medication variables which may influence clinical outcomes. Second, although the study included 1131 patients with COVID-19, severe cases and death cases occurred in a small number of patients, resulting in wider confidence intervals for the ORs and HRs describing associations with these outcomes. Third, we could not construct a risk model of thrombosis. Due to prophylactic anticoagulation, only 13 patients developed thrombosis. The number of patients with thrombosis is not enough to explore the risk factors of thrombosis. On addition, because of the rapid control of the outbreak in China, in a short period, we could not get a validation cohort, which is necessary to evaluate the performance of a risk model of thrombosis. Fourth, the criteria of severe was a retrospective one rather than clinical. The CT tests were not done on all patients to rule out PE, and the vascular ultrasound tests were not done on all patients to rule out VTE as well. Therefore, data from PE and VTE is too small to include separately.

In conclusion, longitudinal coagulation parameters abnormalities are common in COVID-19 patients, and associated with disease severity and mortality. Abnormal peak hospitalization PT, FDP, and D-dimer were associated with a higher mortality of patients with COVID-19. Monitoring coagulation parameters should be advisable to improve the clinical management of patients with COVID-19.
